# The possible role of GABA_A_ receptors and gephyrin in epileptogenesis

**DOI:** 10.3389/fncel.2013.00113

**Published:** 2013-07-22

**Authors:** Marco I. González

**Affiliations:** Division of Neurology and Translational Epilepsy Research Program, Department of Pediatrics, University of Colorado School of MedicineAurora, CO, USA

**Keywords:** epilepsy, epileptogenesis, GABA receptors, gephyrin, status epilepticus

## Abstract

The term epileptogenesis refers to a dynamic alteration in neuronal excitability that promotes the appearance of spontaneous seizures. Temporal lobe epilepsy, the most common type of acquired epilepsy, often develops after an insult to the brain such as trauma, febrile seizures, encephalitis, or status epilepticus. During the pre-epileptic state (also referred as latent or silent period) there is a plethora of molecular, biochemical, and structural changes that lead to the generation of recurrent spontaneous seizures (or epilepsy). The specific contribution of these alterations to epilepsy development is unclear, but a loss of inhibition has been associated with the increased excitability detected in the latent period. A rapid increase in neuronal hyperexcitability could be due, at least in part, to a decline in the number of physiologically active GABA_A_ receptors (GABA_A_R). Altered expression of scaffolding proteins involved in the trafficking and anchoring of GABA_A_R could directly impact the stability of GABAergic synapses and promote a deficiency in inhibitory neurotransmission. Uncovering the molecular mechanisms operating during epileptogenesis and its possible impact on the regulation of GABA_A_R and scaffolding proteins may offer new targets to prevent the development of epilepsy.

This review centers on the possible role of GABA_A_ receptors (GABA_A_R) and the scaffolding protein gephyrin in epileptogenesis. The basic premise is that disruption of the network of proteins involved in the trafficking and anchoring of GABA_A_R might result in decreased inhibitory drive, which may promote the development of spontaneous seizures. A brief introduction to epilepsy and epileptogenesis is provided along with some of the fundamental aspects of the regulation of GABA_A_R by gephyrin. Finally an overview of the alterations in gephyrin and GABA_A_R function observed during epileptogenesis is included.

## EPILEPTOGENESIS AND EPILEPSY

In the majority of patients, temporal lobe epilepsy (TLE) appears to be the result of an injury to the brain caused by trauma, febrile seizures, encephalitis, or status epilepticus (SE; Duncan et al., 2006; Sharma et al., 2007). The precise point in time when spontaneous seizures appear is unknown but it is suspected that following a brain injury there is a pre-epileptic state when many changes occur and transform a normal brain into an epileptic one. Since in many cases, after a brain injury, patients recover from a triggering injury without presenting overt spontaneous seizures, the period that precedes the appearance of recurrent epileptic seizures is known as latent or silent period (Sharma et al., 2007; O’Dell et al., 2012). During the early stages of the chronic period spontaneous seizures are more likely to be controlled with medication but as the disease progress seizures become less responsive to medication and might become intractable (Wieser, 2004). So far there is a consensus that acquired epilepsy results from a triggering insult to the brain and that the cellular and molecular alterations resulting from such insult play a key role in the development of epilepsy, but a direct link between the brain abnormalities detected in epileptic tissue and the generation of recurrent spontaneous seizures remains to be firmly established.

Due to the inherent or obvious limitations associated with the acquisition of human samples, the mechanisms responsible for the appearance of spontaneous seizures are being aggressively studied in experimental models of epilepsy. Post-SE models of chronic epilepsy closely mimic the clinical manifestations and tissue abnormalities observed in humans affected with TLE (Sharma et al., 2007; Curia et al., 2008; Loscher and Brandt, 2010). Induction of SE in rodents by systemic or local administration of a chemoconvulsant is usually followed by a silent (latent) period lasting days or weeks when no obvious seizure activity is observed. During the latent period brain abnormalities develop and inter-ictal activity becomes more frequent, and then suddenly with no apparent cause overt spontaneous seizures manifest (Curia et al., 2008; Scorza et al., 2009). To capitalize the methodological advantages provided by the experimental models, several laboratories have attempted to characterize and establish the duration of the latent (silent) period, unfortunately these attempts have yielded a wide range of measures (Williams et al., 2007). It appears that despite the advantages provided by a well-controlled experimental setting, the animals used in the experiments and the type and severity of injury being tested might impact the length of the latent period. More importantly, some methodological limitations (i.e., accuracy of seizure detection, continuous vs. intermittent monitoring, authentication of when the first seizure occurred, etc.) directly impact the accuracy of the measurements and need to be overcome in order to obtain a definitive characterization of the latent period (Williams et al., 2007).

The dynamic process characterized by progressive alterations in neuronal excitability that promotes appearance of spontaneous seizures and its associated structural lesions is known as epileptogenesis (Pitkanen and Lukasiuk, 2011). Currently, the terms epileptogenesis and latent (or silent) period are used interchangeably to describe the period that encompass the occurrence of an insult to the brain and the appearance epileptic seizures. However, recent findings in patients and experimental models suggest that the alterations resulting from an injury to the brain might progress beyond the appearance of the first spontaneous seizure. Accordingly, it has been suggested that during the natural evolution of epilepsy, each new episode of spontaneous seizures (an insult itself) produces new damage that compounds the damage produced by the original brain injury (Williams et al., 2007; Sloviter, 2008; Pitkanen and Lukasiuk, 2009; O’Dell et al., 2012). Many cellular and molecular alterations in both neuronal and non-neuronal cells have been observed during epileptogenesis. Neuronal alterations include neurodegeneration, neurogenesis, and axonal damage in addition to the architectural reorganization of neuronal processes that result from abnormal sprouting and altered dendritic plasticity. Astrocytes become hypertrophic, develop longer and thicker processes and increase the expression of glial fibrillary acidic protein all as part of a process known as reactive astrogliosis (Gibbons et al., 2012; Heinemann et al., 2012; Kovacs et al., 2012). During epileptogenesis there are many pathological processes that affect brain excitability including the breakdown of the blood–brain barrier (BBB) and inflammation. Ultrastructural analysis of epileptic tissue revealed significant abnormalities in the BBB components and focal opening of the BBB by direct application of albumin can lead to the generation of an epileptic focus (Ivens et al., 2007; Vezzani et al., 2011; Heinemann et al., 2012). Further, the magnitude of BBB leakage occurring during epileptogenesis has been shown to directly correlate with the seizure frequency detected during the chronic period (van Vliet et al., 2007; Vezzani et al., 2013). Inflammatory response due to activation of microglia and astrocytes is associated with brain damage and increased BBB permeability in the tissue adjacent to the region of injury (Vezzani et al., 2011; Marchi et al., 2012). SE-induced inflammation is observed during the epileptogenic period and appears to be reactivated by the occurrence of spontaneous seizures, suggesting that inflammation might have a role in epileptogenesis and promote epileptic activity during the chronic period due to the continuous presence of inflammation intermediaries (Vezzani et al., 2011, 2013).

## GABA_A_ RECEPTORS AND ITS ASSOCIATED PROTEINS

The majority of fast inhibitory neurotransmission in the mature brain is mediated by anion-selective GABA_A_R that are assembled as pentamers from an array of multiple subunit subtypes including α1–2, β1–3, γ1–3, δ, ε, π, θ, and σ1–3 (Fritschy, 2008; Jacob et al., 2008; Luscher et al., 2011; Brickley and Mody, 2012). The subunit composition of GABA_A_R governs the intrinsic properties of the channel such as affinity for GABA, receptor kinetics, conductance, and allosteric modulation. In addition, intracellular loops of each subunit have the potential to interact with scaffolding proteins and affect the cellular distribution and clustering (synaptic or extrasynaptic) of the channels (Jacob et al., 2008; Luscher et al., 2011; Brickley and Mody, 2012). GABA_A_R assembled by combining γ2 and α1–3 subunits (α1–3,βx,γ2) are more commonly located at synaptic sites and mostly responsible for phasic inhibition, whereas receptors located at perisynaptic or extrasynaptic sites are primarily composed of α4 or α6 subunits combined with δ subunits (α4/α6,βx,δ) and mediate most tonic inhibition (Fritschy, 2008; Jacob et al., 2008; Luscher et al., 2011; Hines et al., 2012). Notably, tonic currents in pyramidal neurons of the hippocampus can also be generated by receptors containing α5 subunits (α5βγ2) that are located at extrasynaptic locations (Brickley and Mody, 2012; Hines et al., 2012).

Typically, following synthesis in the endoplasmic reticulum, GABA_A_R are delivered to extrasynaptic compartments within the plasma membrane and then diffuse toward its final destination in either synaptic or extrasynaptic locations. GABA_A_R located at the plasma membrane also transit among different cellular compartments due to internalization and recycling events. Thus, the final number of receptors located at the cell surface is determined by continuous insertion of *de novo* synthesized and recycled receptors (Michels and Moss, 2007; Leidenheimer, 2008). There are a number of accessory proteins that facilitate the transit of GABA_A_R along the biosynthetic pathway and the different recycling compartments within the cell (Chen and Olsen, 2007; Jacob et al., 2008; Leidenheimer, 2008). Early on, during GABA_A_R oligomerization, accessory proteins like BIP (heavy chain binding protein), calnexin and BIG2 (Brefeldin-A-inhibited GDP/GTP exchange factor 2) form interactions with the nascent receptors within the membranous compartments of the endoplasmic reticulum and help with the translocation of the receptors into the Golgi apparatus. During the vesicular trafficking of receptors toward the plasma membrane, GABA_A_R containing γ subunits are linked to tubulin and the microtubules by GABARAP (GABA_A_R-associated protein) that acts as a bridge between vesicles containing GABA_A_R and the machinery that moves those vesicles toward the plasma membrane. GABARAP can also bind to NSF (*N*-ethylmaleimide-sensitive factor), PRIPs (phospholipase-C-related catalytically inactive proteins) and GRIF (GABA_A_R-interacting factors also known as TRAK), and together these proteins promote the interaction of intracellular vesicles containing GABA_A_R with the cytoskeleton and facilitate the motor-dependent transport of receptors toward the plasma membrane (Kneussel et al., 2000; Wang and Olsen, 2000; Charych et al., 2004). The final destination of GABA_A_R into synaptic or extrasynaptic sites is intrinsically determined by the subunits forming the channels and extrinsically by protein–protein interactions with scaffolding proteins (Luscher and Keller, 2004; Chen and Olsen, 2007; Jacob et al., 2008; Leidenheimer, 2008).

Gephyrin is the main structural scaffold that links proteins located at the subsynaptic compartment with the cytoskeleton and it is required for the organization and clustering of GABA_A_R at inhibitory synapses (Michels and Moss, 2007; Fritschy et al., 2008). During development, increased concentration of gephyrin precedes the accumulation of GABA_A_R at synaptic sites and facilitates the formation and stabilization of inhibitory synapses (Christie et al., 2002; Danglot et al., 2003; Swanwick et al., 2006; Fritschy et al., 2008). Removal of gephyrin by gene targeting or siRNA interference strongly affects GABA_A_R clustering and reduces inhibitory post-synaptic currents, a reciprocal effect on gephyrin clustering and GABAergic innervations has been observed following elimination of γ2 subunits (Essrich et al., 1998; Kneussel et al., 1999; Li et al., 2005; Yu et al., 2008). GABA_A_R clustering also occurs in neurons lacking gephyrin, but the clusters formed in these conditions have increased mobility and show less accumulation at inhibitory synapses, which reinforces the notion that gephyrin enhance anchoring of receptors at synaptic sites (Levi et al., 2004; Jacob et al., 2005; Yu et al., 2007). GABA_A_R containing α5 subunits are responsible for tonic inhibition and can also be found forming extrasynaptic clusters (Brunig et al., 2002; Loebrich et al., 2006). A crucial player in α5-containing GABA_A_R clustering is radixin. Both radixin antisense and genetic knockout causes a loss of α5 clusters in hippocampal neurons and hippocampal tissue (Loebrich et al., 2006; Kneussel and Loebrich, 2007). Radixin and gephyrin do not interact or colocalize with each other, suggesting that the mechanisms behind radixin and gephyrin-dependent clustering are independent (Loebrich et al., 2006; Kneussel and Loebrich, 2007). 

The structural and molecular details underlying the regulation of GABA_A_R by gephyrin are starting to emerge. Gephyrin consist of three major domains, a 20-kDa N-terminal domain (G-domain), a 43-kDa C-terminal domain (E-domain), and an 18–21 kDa central domain (C-domain; Saiyed et al., 2007; Fritschy et al., 2008). Trimerization of the G-domain and dimerization of the E-domain appear to be the oligomerization pattern involved in the formation of the hexagonal lattice required for proper formation of inhibitory synapses (Sola et al., 2004; Saiyed et al., 2007; Fritschy et al., 2008; Tyagarajan and Fritschy, 2010; Herweg and Schwarz, 2012). In addition to its role in the formation of the gephyrin lattice, the E-domain is the binding site for GABA_A_R (Tyagarajan and Fritschy, 2010) whereas the C-domain binds to several accessory proteins like Pin1 (Zita et al., 2007), dynein light chain 1 and 2 (Fuhrmann et al., 2002; Maas et al., 2006) and collybistin (Kins et al., 2000). Experiments in recombinant expression systems have demonstrated that collybistin is essential for the distribution and stabilization of gephyrin at post-synaptic sites and that overexpression of selected collybistin domains increases the size and density of gephyrin clusters (Kins et al., 2000; Chiou et al., 2011; Tyagarajan et al., 2011), suggesting that collybistin aids with the proper clustering of GABA_A_R at synaptic sites by forming a partnership with gephyrin (Yu et al., 2008). The small GTPase Cdc42 also binds to the C-domain of gephyrin and works in collaboration with collybistin to help with the translocation of gephyrin toward the plasma membrane (Tyagarajan et al., 2011). Another protein that interacts with gephyrin and indirectly regulates the post-synaptic trafficking and/or accumulation of GABA_A_R is termed GRIP1 (glutamate receptor interacting protein 1). *In vivo* and *in vitro* evidence suggest that GRIP1 is located at GABAergic synapses and physically interacts with gephyrin to directly modulate gephyrin clustering and indirectly regulate the post-synaptic distribution of GABA_A_R (Yu et al., 2008).

## ALTERATIONS IN GABA_A_R AND SCAFFOLDING PROTEINS DURING EPILEPTOGENESIS

The belief that following a brain injury there is a quiescent, pre-epileptic state in which there is gradual changes at the molecular, cellular, and circuit levels that ultimately results in the manifestation of spontaneous seizures, has led to the search for mechanisms underlying epileptogenesis. Induction of SE using the chemoconvulsant pilocarpine produces a transient decrease in GABAergic drive readily detectable during the latent period. Abnormal electroencephalogram (EEG) patterns, such as large amplitude spikes and sharp waves can be detected as early as 3–5 days following SE, which overtime culminate with the appearance of full-blown electrographic seizures (El-Hassar et al., 2007). During SE there is a rapid increase in neuronal hyperexcitability due to a quick decline in the number of physiologically active GABA_A_R at the plasma membrane (Goodkin et al., 2005; Naylor et al., 2005). SE triggers a rapid loss of synaptic GABA_A_R containing β and γ subunits while extrasynaptic receptors containing α5 and δ subunits remain unaffected (Goodkin et al., 2008; Terunuma et al., 2008). A decrease in the phosphorylation of β3 subunits allows the interaction of β3-containing GABA_A_R with the clathrin-adaptor protein 2 and the recruitment of GABA_A_R into clathrin-coated pits promotes a faster removal of these receptors from the cell surface, suggesting that a decrease in the phosphorylation of β3 subunits may account for the selective loss of synaptic GABA_A_R observed following SE (Goodkin et al., 2008; Terunuma et al., 2008). These biochemical observations directly link the decrease in miniature inhibitory post-synaptic currents observed after induction of SE with the selective internalization of synaptic GABA_A_R containing β and γ subunits and explain why the currents mediated by extrasynaptic receptors are spared (Goodkin et al., 2005; Naylor et al., 2005).

**FIGURE 1:**
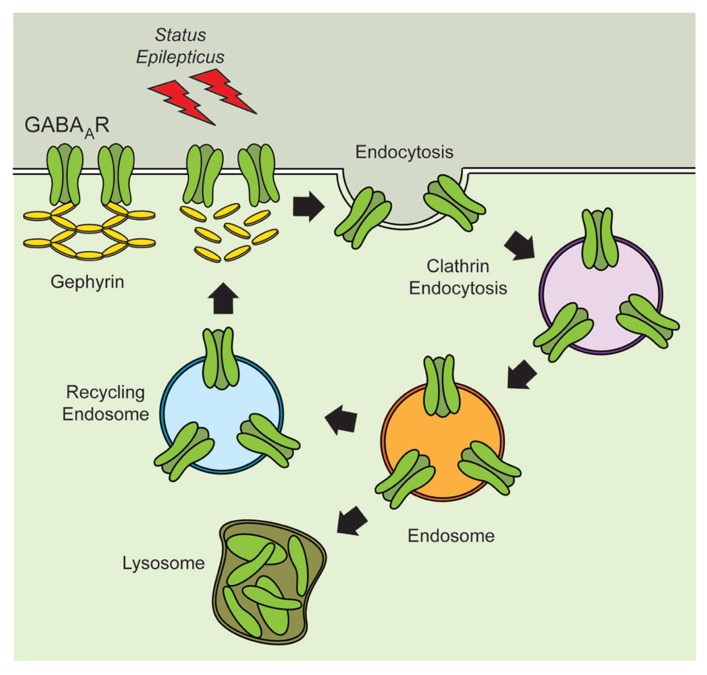
** Altered stability of GABA_A_R and scaffolding proteins during epileptogenesis.** Induction of *status epilepticus* produces alterations in the expression of gephyrin and might disrupt GABA_A_R anchoring. Decreased expression of gephyrin might compromise the recycling of GABA_A_R and reduce the stability of receptors present at the plasma membrane. Reduced expression of GABA_A_R at the plasma membrane may contribute to the increased excitability observed during epileptogenesis.

The fate of internalized receptors following induction of SE is more likely to be determined by the cellular compartment where they are transiently stored (**Figure 1**). Receptors present in endosomal compartments can be reincorporated into the active pool of receptors at the plasma membrane or they can be relocated to the lysosomes for degradation (Chen et al., 2007; Wasterlain and Chen, 2008). Recent studies suggest that the network of proteins required for the proper trafficking and anchoring of GABA_A_R might be disrupted following SE. During the latent period there is a reduction in the total expression of gephyrin that appears to translate into a reduction in the number of gephyrin clusters (Knuesel et al., 2001; Thind et al., 2010; Fang et al., 2011; González et al., 2013). The pattern of gephyrin loss observed during the silent period parallels the changes in excitability previously observed, and suggests that the loss of scaffolding proteins directly impact the function of GABA_A_R during epileptogenesis. Intriguingly, during the chronic period there is an increase in both the total expression and the number of gephyrin clusters (Thind et al., 2010; Fang et al., 2011), but it is unclear if this rebound in gephyrin expression results in fully functional inhibitory synapses.

Alterations in the expression of GABA_A_R during the epileptogenic period include rapid down-regulation of α4, β2/3, γ2, and δ subunits (Schwarzer et al., 1997; Houser and Esclapez, 2003; Peng et al., 2004). Our recent characterization of the expression of several GABA_A_R subunits in microdissected CA1 also showed a reduction in the levels of α4, β2/3, and γ2 (but not α1) subunits as early as 4 days after SE (González et al., 2013). The loss of these subunits correlated with the down-regulation of gephyrin, suggesting that the loss of GABA_A_R might result from the lack of proper receptor anchoring and clustering (González et al., 2013). Accordingly, analysis of the cell surface levels of GABA_A_R revealed a time-dependent reduction in the plasma membrane levels of α4 and γ2 subunits that correlated with the down-regulation of gephyrin (González et al., 2013). These observations hint to the possibility that during the epileptogenic period, the stability of the GABA_A_R receptors that recycle back to the plasma membrane might be compromised because they cannot be properly anchored (González et al., 2013). They also suggest that the loss of inhibition and increased inter-ictal activity observed during the latent period might result from the persistent dysregulation of GABA_A_R trafficking and anchoring (Wasterlain and Chen, 2008). However, whether a loss of scaffolding proteins is a factor contributing to the hyperexcitability observed during the epileptogenic period remains to be fully characterized.

Additional support for hypothesis that a lack of GABA_A_R stability contributes to the hyperexcitability observed during the latent period comes from studies in *Xenopus* oocytes transplanted with cell membranes isolated from epileptic tissue (Palma et al., 2007; Mazzuferi et al., 2010). Repetitive stimulation of microtransplanted receptors induces a characteristic run-down of GABA_A_R-mediated currents that is independent of changes in receptor affinity or membrane potential (Palma et al., 2007). The run-down in GABA_A_R currents can be readily detected in receptors isolated following the manifestation of the first spontaneous seizure and its appearance has been associated with the transition from the latent to the chronic stage of epilepsy (Mazzuferi et al., 2010). Initial exploration of the molecular mechanisms behind this phenomenon revealed a switch in the composition of GABA_A_R and uncovered an increase in the ratio of α4/α1 subunits incorporated into receptors. More importantly, the switch in GABA_A_R assembly occurs at the same time that the current run-down appears, underscoring previous findings showing alterations in the expression of α4 and α1 subunits that affect the assembly, localization, and function of GABA_A_R and results in the impairment of tonic and phasic inhibition (Brooks-Kayal et al., 1998; Peng et al., 2004; Mazzuferi et al., 2010).

The specific mechanisms involved in the regulation of gephyrin during epileptogenesis and epilepsy remain to be fully characterized, but some clues are starting to emerge. Analysis of samples obtained from epileptic patients show a reduction in gephyrin expression, which correlates with the appearance of protein fragments probably resulting from gephyrin degradation (Forstera et al., 2010; Fang et al., 2011). The process involved in the generation of gephyrin fragments remains unclear but a favored hypothesis is that cellular stress (alkalosis and hyperthermia) might be sufficient to induce the skipping of exons in gephyrin messenger RNA resulting in the production of abnormally spliced variants of gephyrin. These abnormal variants may then interact with normal gephyrin molecules and act as dominant-negative mutants and promote the accumulation of gephyrin in ubiquitin positive inclusions (Forstera et al., 2010). The impact abnormally spliced variants of gephyrin has been associated with oligomerization deficits and aberrant clustering of GABA_A_R containing α2 subunits (Forstera et al., 2010). Induction of mild seizures or inflammatory events triggers the generation of adult-born hippocampal neurons and increases the expression of gephyrin, mostly in the newly generated neurons (Jakubs et al., 2006, 2008; Jackson et al., 2012). Electrophysiological recordings revealed that newborn neurons produced after these injuries have reduced excitatory and increased inhibitory drive partially associated with the increase in gephyrin expression. Together, these observations point out to the possibility that newly generated cells might increase gephyrin expression as a compensatory mechanism to mitigate the hippocampal hyperexcitability observed after an insult to the brain (Jakubs et al., 2006; Jackson et al., 2012).

Another line of evidence implicating gephyrin dysfunction as a key element in the generation of epileptic seizures includes recent genetic evidence found in individuals affected by pathologies associated with a seizure phenotype. Rare hemizygous microdeletions in the chromosome 14q23.3 that encompass exons 3–5 in the coding region the G-domain were found in individuals from six unrelated families presenting autism, schizophrenia, and seizures (Lionel et al., 2013). Other mutations that interfere with collybistin function have also been associated with abnormalities observed in patients with epilepsy and mental retardation (Harvey et al., 2004; Marco et al., 2008; Kalscheuer et al., 2009; Lesca et al., 2011). These mutations found in the coding sequence of collybistin interfere with the somatic and synaptic localization of gephyrin via dominant-negative mechanisms that indirectly affect the distribution and synaptic clustering of GABA_A_R (Harvey et al., 2004; Kalscheuer et al., 2009).

The impact that disruption of gephyrin and other scaffolding proteins might have on GABA_A_R function during epileptogenesis remains to be elucidated. If a loss of gephyrin directly impacts the number and function of GABA_A_R at inhibitory synapses, interventions to promote the stability of gephyrin and GABA_A_R might ameliorate the deleterious changes in excitability observed during epileptogenesis and epilepsy. Altered expression of gephyrin has been observed in several pathologies presenting symptomatic seizures, but it is unclear if changes in gephyrin are beneficial or pathologic (Jakubs et al., 2008; Thind et al., 2010; Jackson et al., 2012). Understanding the molecular mechanism(s) behind the dysregulation of scaffolding proteins involved in the regulation of GABA_A_R might provide new insights into the pathologic events that contribute to the generation of spontaneous seizures and might offer new targets to disrupt epileptogenesis and prevent epilepsy.

## Conflict of Interest Statement

The author declares that the research was conducted in the absence of any commercial or financial relationships that could be construed as a potential conflict of interest.
